# Commentary: Factors Associated With Non-participation in Cohort Studies Emphasize the Need to Generalize the Results With Care

**DOI:** 10.2188/jea.JE20140269

**Published:** 2015-02-05

**Authors:** Motoki Iwasaki

**Affiliations:** Division of Epidemiology, Research Center for Cancer Prevention and Screening, National Cancer Center, Tokyo, Japan

Epidemiologic studies with a high proportion of non-participants can suffer from selection bias and the limited generalizability of their results. The determinants of non-participation in an epidemiologic study should be assessed, and the results might help establish effective strategies to increase participation in future studies.

Hara and colleagues investigated factors associated with non-participation in a face-to-face second survey conducted 5 years after the baseline survey in one study area of the Japan Multi-Institutional Collaborative Cohort Study (J-MICC).^1^ The study was comprehensive and well organized: information from the baseline questionnaire was compared between participants and non-participants in the face-to-face second survey, and the self-reported incidence of disease during the 5-year follow-up was compared between participants and non-participants by a mail and telephone health survey of the non-participants. Factors from the baseline questionnaire associated with non-participation were female sex, youngest and oldest ages, lower education, lower occupational class, current smoking, lower physical activity level, shorter sleep time, obesity, and constipation. Interestingly, participants who developed cancer during the follow-up period were less likely to participate in the second survey. Having found that some factors associated with non-participation are risk factors for cancer and that cancer occurrence during the follow-up was associated with non-participation, the authors sought to promote awareness of bias due to non-participation.

The two aims of a follow-up (second) survey in a cohort study are exposure assessment and identification of disease occurrence during the follow-up period. This second aim is hindered by a high proportion of non-participants, which results in incomplete follow-up and possible distortion of the exposure-disease association. Thus, a range of efforts to increase response in the follow-up survey should be made. Examples can be seen in the Nurses’ Health Study and Health Professional Follow-up Study; these studies established a follow-up system based on postal mailing and achieved high response rates.^2^ If such strategies are not possible, identification of disease occurrence should rely on a disease registry that covers the study population. For exposure assessment, the distribution of some exposures differs between participants and non-participants, as Hara et al showed. In general, participants tend to be highly motivated and health-conscious. If the proportion of non-participants is high, the prevalence of exposure and absolute risk of the outcome in a cohort study will differ between the study participants and target population from which they were derived, limiting generalizability. In contrast, relative risk (RR) estimates are considered more robust measures for generalizability than the prevalence of exposure and absolute risk of the outcome; however, few studies have actually explored potential effects of non-participation on RR estimates.

Iwasaki and colleagues demonstrated a practical example of the degree of generalizability of RR estimates using data from a large population-based prospective cohort study, the Japan Public Health Center-based Prospective (JPHC) Study.^3^ Study subjects were defined as all residents whose addresses were registered in 27 municipalities that were supervised by 9 public health centers at baseline. In the baseline survey, a total of 45 452 men (79%) returned the self-administered questionnaire, 12 162 (26.8%) of whom provided health check-up data. Since health check-up examinees were a subset of the respondents to the baseline questionnaire, the respondents were considered to represent the general population (population), and the health check-up examinees were considered to represent a sample from a well-defined population (sample). Using two different datasets—the population (*n* = 45 452) and the sample (*n* = 12 162)—associations of smoking status and BMI with all-cause mortality were examined, and confounder-adjusted RRs in the population and sample were compared using empirical sampling distributions from the population. Although current smokers showed a significantly increased risk of all-cause mortality for both the population and the sample, adjusted RRs of current smokers were significantly higher in the sample (RR 1.83) than in the population (RR 1.48). Regarding the association between BMI and the risk of all-cause mortality, a U-shaped curve was observed in the population: compared with the BMI categories of 23.0–24.9, statistically significant RRs were found in the lowest three underweight categories and the highest overweight category in the population. In contrast, no statistically significant association was observed in the sample. In particular, adjusted RR in the lowest underweight category (BMI of 14.0–18.9) was significantly lower in the sample (RR 1.30) than in the population (RR 2.06). These findings are only one example, but clearly show that the adjusted RRs significantly differed between the sample and the population and either under- or over-estimated the associations for some categories of the sample. This indicates that the RR estimates for a well-defined population cannot necessarily be “generalized” to the general population.

Because of feasibility, cohort studies are commonly conducted using samples from well-defined populations, such as a defined group of workers (eg, nurses or civil servants) or groups of individuals involved in health-related programs (eg, health check-up or health care insurance). At the very least, these samples can provide valid RR estimates for a well-defined population. However, extrapolation to the general population is not guaranteed, and relies instead on common underlying biological mechanisms of disease development. Although the above examples by Iwasaki et al represent only a single refutation of the broadly expected hypothesis on the generalizability of RRs, this problem might influence not only studies using well-defined populations but also those with a relatively low proportion of participants and represents a common issue for cohort studies. In fact, only 12 078 (19.7%) of 61 447 residents aged 40 to 69 years old agreed to participate in the baseline survey of the J-MICC study Saga region,^4^ and even among these baseline participants, only 8454 (73.6%) participated in the second survey.^1^

For these reasons, the generalizability of RR estimates should be carefully and cautiously evaluated, with due consideration to the intended application of the findings. One major application is that RR estimates are used to assess causality as an indicator of the strength of association. In these cases, establishing causality requires consistent findings from several high-quality studies. A second application is that RR estimates are used to calculate population-attributable fractions, which contribute to evidence-based policy decision-making for disease control. In these cases, further cautious evaluation is needed because of the use of population-specific values.
